# HPLC Purification of TRPM8 and Experimental Confirmation of Its Cholesterol Affinity on Synthetic Lipid Raft-like Models

**DOI:** 10.3390/life16030392

**Published:** 2026-02-28

**Authors:** Clotilde Beatrice Angelucci, Annalaura Sabatucci, Alexandrine Kurtz, Davide Laurenti, Beatrice Dufrusine, Enrico Dainese, Antonio Francioso

**Affiliations:** 1 Department of Veterinary Medicine, University of Teramo, 64100 Teramo, Italy; bcangelucci@unite.it; 2 Department of Bioscience and Technology for Food, Agriculture and Environment, University of Teramo, 64100 Teramo, Italy; alsabatucci@unite.it (A.S.);; 3 Department of Biochemical Sciences “A. Rossi Fanelli”, Sapienza University of Rome, 00185 Rome, Italy

**Keywords:** TRPM8, ion channel, cholesterol, FRET, HPLC-SEC, lipid rafts

## Abstract

This study presents the successful expression, purification, and functional characterization of the human TRPM8 ion channel, a key player in temperature sensing and pain modulation. Using a modified bacterial expression protocol and DDM-based solubilization, TRPM8 was purified via HPLC-SEC and analyzed for its membrane-binding properties. FRET-based assays with synthetic lipid rafts revealed a strong and selective affinity of TRPM8 for cholesterol-containing membranes, suggesting cholesterol’s role in modulating TRPM8 localization and activity. These findings provide quantitative in vitro evidence of TRPM8–cholesterol interactions and establish a robust model system for future structural and functional studies of membrane-associated proteins.

## 1. Introduction

The Transient Receptor Potential Melastatin 8 (TRPM8) represents one of the most fascinating ion channels in mammalian biology, standing at the intersection of temperature sensing, pain perception, and various physiological processes. First discovered in 2002 through independent studies [1,2], this remarkable protein has emerged as a crucial molecular sensor that fundamentally shapes how organisms perceive and respond to their thermal environment [3].

Encoded by the TRPM8 gene (Chromosome 2), the canonical sequence of hTRPM8 consists of 1104 residues, for a molecular weight of 128 kDa. At its core, TRPM8 is an architecturally complex protein that assembles as a tetramer in cell membranes. Each monomer contributes six transmembrane domains to the channel structure, with the fifth and sixth domains forming the central ion-conducting pore [4,5]. The protein’s sophisticated architecture includes extensive cytoplasmic regions at both the N- and C-termini, which play crucial roles in channel regulation and cellular signaling. This structural sophistication enables TRPM8 to perform its remarkable function as a molecular cold sensor, activating when temperatures drop below approximately 26 °C.

The channel’s activation mechanism represents a marvel of molecular engineering. TRPM8 responds not only to cold temperatures but also to various chemical compounds that evoke cooling sensations. Menthol, the compound responsible for the refreshing sensation of peppermint, serves as a primary agonist, binding to specific sites within the channel’s structure to promote opening. This dual activation by both physical (temperature) and chemical stimuli demonstrates the channel’s remarkable versatility in environmental sensing [3].

In terms of its distribution throughout the body, TRPM8 exhibits a carefully orchestrated expression pattern that aligns with its physiological roles. The channel is most abundantly expressed in sensory neurons within the dorsal root and trigeminal ganglia, where it serves as a primary cold sensor. However, its presence extends beyond these neural tissues to include various other cell types, including prostate epithelial cells, bladder urothelium, and specific populations of arterial smooth muscle cells. This diverse distribution pattern hints at the channel’s multifaceted physiological roles beyond simple temperature sensing.

The physiological impact of TRPM8 extends far beyond its well-known role in cold perception. When activated, the channel allows calcium and sodium ions to flow into cells, triggering a cascade of cellular responses that ultimately influence various biological processes. In sensory neurons, this ionic flux translates environmental temperature information into electrical signals that the brain interprets as cold sensation. However, the channel’s influence extends to numerous other physiological processes, including thermoregulation, pain modulation, and cellular proliferation.

TRPM8’s involvement in pathological conditions has drawn significant attention from the medical research community. The channel plays crucial roles in various pain conditions, particularly those involving cold hypersensitivity and neuropathic pain. Its expression patterns become altered in several cancer types, notably prostate cancer, where it may influence tumor progression and survival [6,7] or colorectal cancer, where it could be used as a prognostic marker [8]. These pathological associations have positioned TRPM8 as a promising therapeutic target, spurring extensive drug development efforts aimed at modulating its activity.

The purification and characterization of TRPM8’s membrane interactions, particularly with cholesterol, represents a complex endeavor in protein biochemistry that has provided crucial insights into channel function and regulation.

The extraction of TRPM8 from cellular membranes represents a critical step that requires careful optimization of conditions to maintain protein stability and functionality. The choice of detergent proves particularly crucial, with n-Dodecyl β-D-maltoside (DDM) [9] and Lauryl Maltose Neopentyl Glycol (LMNG) [10] emerging as preferred options due to their ability to maintain protein stability while effectively solubilizing membrane components.

The interaction between TRPM8 and cellular membranes extends far beyond simple integration of its transmembrane domains. The channel displays remarkable sensitivity to membrane composition, with cholesterol emerging as a key modulator of channel function. The presence of multiple cholesterol recognition/interaction amino acid consensus (CRAC/CARC) motifs within the TRPM8 structure suggests evolved mechanisms for specific cholesterol sensing. These motifs, characterized by specific amino acid sequences, are strategically positioned within the channel’s architecture to facilitate interaction with membrane cholesterol [11].

The functional significance of TRPM8–cholesterol interaction extends to the channel’s distribution and organization within the membrane. Evidence suggests that cholesterol content influences the channel’s localization to specific membrane microdomains, or lipid rafts, which in turn affects its accessibility to various regulatory molecules and its ability to participate in signaling cascades. This organization appears particularly important for the channel’s role in cold sensation and pain signaling, where precise control of channel activity is crucial for appropriate physiological responses, but a role has been suggested for this receptor also in cholesterol-mediated neuronal cell dynamics modulation in neurodegenerative diseases [12].

The experimental evidence for direct TRPM8–cholesterol interactions using lipid rafts is relatively limited, and no studies using FRET techniques confirmed the direct experimental binding of the isolated protein to cholesterol. In this work we expressed and purified TRPM8 protein and we used Large Unilamellar Vesicles (LUVs) as synthetic lipid rafts for the study of TRPM8 affinity to cholesterol via a FRET-based approach.

The protein purified by HPLC-SEC chromatography and FRET experiments demonstrated higher affinity for cholesterol-containing synthetic lipid rafts. The use of this model system to study membrane interactions with cholesterol represents and validates an excellent model for future structural and functional studies on TRPM8 and other analogous proteins with lipophilic membrane ligands.

## 2. Materials and Methods

All reagents were of analytical grade and were purchased from Merck, unless otherwise stated.

### 2.1. Protein Expression and Purification

The protocol for hTRPM8 expression from bacteria was based on the one published by Zakharian et al. for rat TRPM8 [9], with slight modifications.

Briefly, Chemically Competent *E. coli* One Shot™ BL21 Star™ (DE3) cells (Invitrogen, Waltham MA, USA) were transformed with pET-21a(+) vector (Novagen, Darmstadt, Germany) containing the N-6xHis-hTRPM8 coding sequence for the canonical isoform of the protein, namely isoform 1 (UNIPROT ID Q7Z2W7-1). The transfected bacteria were grown in LB medium in the presence of 100 µg/mL ampicillin at 37 °C. Recombinant hTRPM8 expression was induced by addition of 1 mM IPTG (isopropyl-D-thiogalactopyranoside). Induction at 37 °C was carried out overnight. After induction, cells were pelleted by centrifugation at 4000× *g* for 20 min two times to remove the medium and washed with PBS. Pellets were conserved at −80 °C until use. Pelleted cells were resuspended in 20 mM Tris-HCl pH 7.5, 10 mM EDTA, 0.1% Triton X-100 with the addition of 1 mM PMSF, 20 µg/mL lysozyme, 20 µg/mL DNase I from bovine pancreas, and 20 µg/mL RNase from bovine pancreas, and lysed by ultrasonic disintegration. Inclusion bodies (IBs) were isolated from lysed cells in 20 mM Tris-HCl pH 7.5, 10 mM EDTA, 1% Triton X-100, and then washed twice in the same buffer, collecting the pellet by centrifugation at 10,000× *g* for 15 min.

The last pellet of the IB was resuspended in LCB buffer, containing (400 mM LiCl, 1 mM MgCl_2_), 15% glycerol, 20 mM HEPES, 0.5% DDM (Avanti Polar Lipids) pH 7.5, and protease-inhibitor mixture tablets (Complete Mini, Roche, Basel, Switzerland). hTRPM8 solubilization was performed with gentle agitation for 2 h at 4 °C.

### 2.2. Gel Electrophoresis and Western Blot Analysis

Proteins (50 µg/well) were electrophoretically separated with 8% sodium dodecyl sulfate–polyacrylamide gel electrophoresis (SDS-PAGE) using Tris-glycine SDS buffer at a constant voltage of 200 V. Precision Plus Protein™ Kaleidoscope™ (Bio-Rad, Hercules, CA, USA) were used as protein standards. The gel was stained with Coomassie Brilliant Blue. Protein was transferred to nitrocellulose membranes in Tris-glycine buffer pH 8.3 containing 20% methanol at 30 V overnight. The membrane was blocked in TNT-milk (15 mM Tris buffer pH 8, 140 mM NaCl, 0.05% Tween 20 and 5% non-fat dry milk) at 4 °C for 3 h, then incubated with primary rabbit monoclonal anti-TRPM8 antibody (code: MA5-35474, Thermo Fisher Scientific, Waltham, MA, USA) in 3% TNT-milk at 4 °C overnight. After rinsing three times in TNT for 10 min each time, the membrane was incubated with anti-rabbit-IgG horseradish peroxidase-linked secondary antibodies (Invitrogen), diluted in 3% TNT-milk (1/20,000) for 1 h. After three 10 min washes in TNT, the membranes were processed for chemiluminescent detection using Pierce™ ECL Plus Western Blotting Substrate (Thermo Fisher Scientific Inc., Waltham, MA, USA) according to the manufacturer’s instructions. The image was developed with the c400 Western Blot Imager (Azure Biosystem, Sierra Court, Suite A-B, Dublin, CA, USA).

### 2.3. HPLC-SEC (Size Exclusion Chromatography)

HPLC-SEC was performed on a series 200 system (Perkin Elmer, Shelton, CT, USA) using a BioSep SEC-s3000 column (4.6 cm × 30 cm) (Phenomenex) with an exclusion range of 5–700 kDa for native proteins. TRPM8 was eluted in LCB buffer not containing DDM detergent, at a flow rate of 0.2 mL/min. Protein elution was monitored by recording the absorbance at the wavelength of 280 nm. Injection volume: 50 uL Fractions of 0.2 mL were collected at 1 min intervals (see Figure 2) Column calibration was performed with 3 different protein standards, namely BSA, y-globulin, and thyroglobulin (Merck KGaA, Darmstadt, Germany) at the concentration of 1 mg/mL.

### 2.4. Synthetic Lipidic Membranes Preparation

All lipids were purchased from Merck (Merck KGaA, Darmstadt, Germany). Large Unilamellar Vesicles (LUVs) in the presence or in the absence of the fluorescent probe 18:1 Pyrene PE (PyPE) 1% (*w*/*v*) were prepared according to Angelucci et al. [13] by the hydration method. LUV size uniformity was achieved using 200 nm polycarbonate filters in the LIPOSOFAST (Avestin, Inc. Ottawa, ON, Canada) extruder system. Two different LUVs compositions were tested at a final lipid concentration of 2 mM: sphygomyelin:cholesterol: 1,2-dioleoyl-sn-glycero-3-phosphocholine (DOPC) (molar ratio 1:1:1) or sphyngomyelin:DOPC (molar ratio 1:1).

### 2.5. FRET Experiments

Protein-membrane affinity measurements were performed by FRET in an F-2710 spectrofluorometer (Hitachi, Tokyo, Japan) according to the protocol published by Angelucci et al. [13]. In brief, FRET measurements use as energy donors the protein tryptophan residues with the emission maximum from 307 to 353 nm and as acceptors the PyPE fluorophore embedded in the LUVs with excitation maximum at 351 nm. The membrane affinity of TRPM8 for LUVs in all the considered experimental conditions was determined through non-linear regression analysis of the fluorescence emission intensity according to the following equation as a function of lipid concentration [*L*].

(1) $$∆F=\frac{{∆F}_{max}\;\left[L\right]}{{\left[L\right]}_{1/2}+\left[L\right]}$$

where Δ*F* = *F*_0_ − *F*, being *F*_0_ the fluorescence intensity at zero lipid concentration.

This allowed us to calculate the [*L*]_1/2_ parameter, that is, the concentration of lipid vesicles at half saturation, a measure of the affinity of the protein to the membrane, proportional to the K_m_. Binding isotherms were analyzed using the Michaelis Menten model equation by means of Past 5 software [14]. hTRPM8 was used at a final concentration of 0.2 μM, whereas the liposome concentration varied between 0 and 305 mM. Fluorescence spectra were recorded at 25 °C, the excitation wavelength was set to 292 nm, while the fluorescence emission spectra were recorded in the 300 to 420 nm range. The quenching of the Fluorescence Intensity (FI) at the maximum emission wavelength of tryptophans was recorded at 335 nm as a function of PyPE-containing LUVs concentration.

Statistical analysis was performed with RStudio v.2025.05.0+496 [15] applying Welch’s *t*-test.

## 3. Results

**Figure 1:**
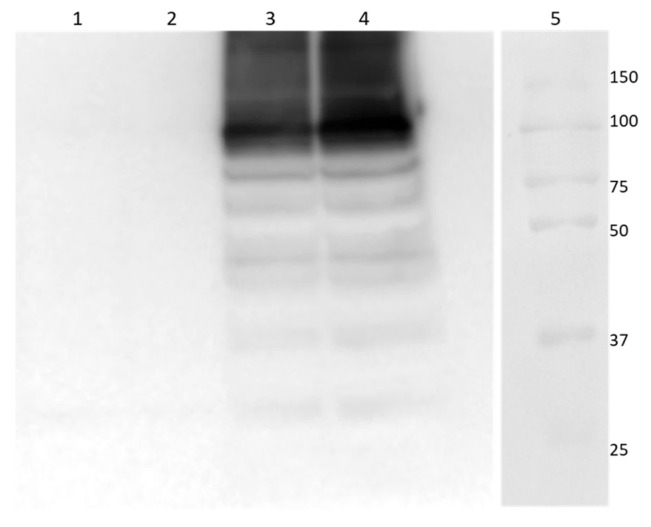
Western Blot of *E. coli* lysed samples. Controls (lanes 1, 2) are cells transfected with a plasmid not containing TRPM8. Samples (lanes 3, 4) are lysates from cells transfected with the pET21+ plasmid containing N-6xHis-hTRPM8. Ladder Precision Plus Protein™ Kaleidoscope™ (lane 5).

In this study, we successfully expressed and purified human TRPM8 (hTRPM8) from *E. coli* using a modified protocol based on Zakharian et al. [9]. As shown by Western Blot analysis (Figure 1), the hTRPM8 main band has a molecular weight around 100 kDa, fully compatible with an hTRPM8 monomer.

**Figure 2:**
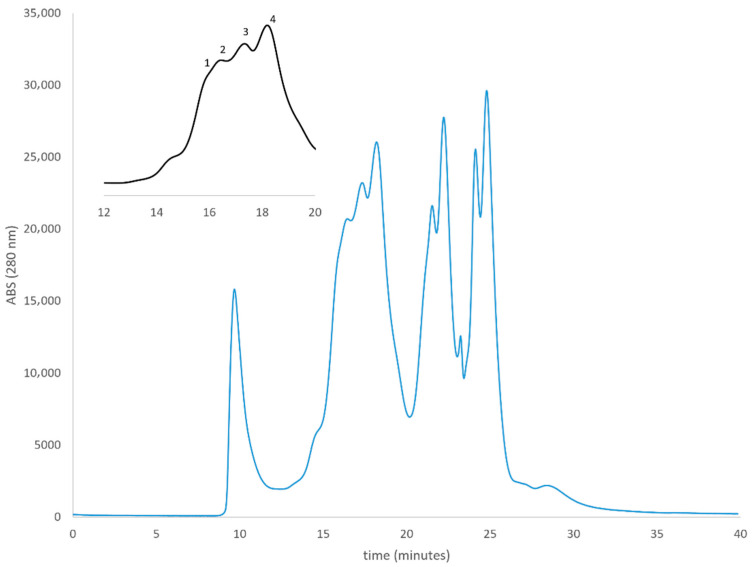
HPLC-SEC chromatogram of hTRPM in the column exclusion range as determined from calibration curve. Four different fractions were collected (namely 1, 2, 3, 4) at 1 min. intervals (inset).

To assess the oligomeric state of the expressed protein, we performed native polyacrylamide gel electrophoresis (Native-PAGE) followed by Western blot analysis (Supplementary Material, Figure S1). Three immunoreactive bands were detected. Two distinct species were resolved at apparent molecular weights of approximately 250 kDa and 150 kDa. A higher molecular-weight component was observed near the top of the gel, suggesting the presence of higher-order complexes exceeding the resolving capacity of the matrix. After expression, the protein solubilized with DDM detergent was purified via HPLC-SEC (Figure 2), yielding four distinct fractions with apparent molecular weights ranging from 49 to 145 kDa (Table 1).

The fraction corresponding to approximately 109 kDa is consistent mainly with the expected size of the hTRPM8 monomer and was further characterized for its membrane-binding properties.

**Figure 3:**
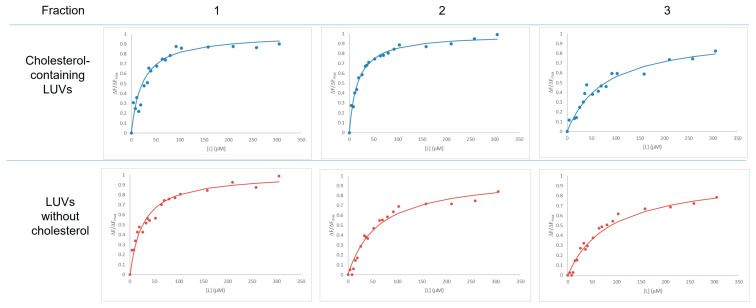
Most representative binding isotherms of the hTRPM8 HPLC-SEC collected fractions to two different LUV compositions. All curves were normalized to the asymptotic parameter of fluorescence emission maximum (ΔF_max_) (see Equation (1)).

To investigate the interaction of hTRPM8 with lipid membranes, we employed a FRET-based assay [13] to determine the affinity of the purified proteins for lipid raft (LR)-like model membranes. To this purpose, we utilized Large Unilamellar Vesicles (LUVs) composed of DOPC, sphingomyelin and cholesterol at equimolar ratios, and a lipid composition mimicking the one found in LRs [16]. As a negative control, LUVs not containing cholesterol were tested (Figure 3).

As reported in Table 2, our results demonstrate a clear preference of hTRPM8 for cholesterol-containing membranes. Specifically, the monomeric fraction (fraction 2) exhibited a significantly lower L½ value (20.6 ± 4.8 µM) for cholesterol-containing LUVs compared to cholesterol-free membranes (62.3 ± 15.1 µM), indicating a higher binding affinity in the presence of cholesterol.

This selective affinity suggests that cholesterol may act as a modulator or facilitator of TRPM8 membrane association, potentially stabilizing the protein within lipid raft-like domains. These findings are in line with previous observations that, like other members of the TRP family [17], TRPM8 localizes to cholesterol-rich microdomains in cellular membranes [18].

Our study provides the first quantitative in vitro evidence of this interaction using purified human isoform and synthetic membranes.

## 4. Discussion and Limitations

In this study, we investigated the association of purified human TRPM8 with cholesterol-containing lipid membranes using size-exclusion chromatography (SEC) and FRET-based assays with lipid raft-like LUVs. Our results indicate that fraction 2, enriched in monomeric TRPM8, consistently shows preferential association with membranes containing cholesterol, providing quantitative biochemical and biophysical evidence that TRPM8 interacts with sterol-containing bilayers. These observations complement previous cellular and structural studies, confirming that even monomeric TRPM8 retains intrinsic affinity for cholesterol-enriched membranes.

Interestingly, the [L]_1/2_ of TRPM8 for cholesterol-containing LUVs are comparable to the values that we already reported for purified membrane-binding enzymes like lipoxygenases or fatty acid hydrolase (FAAH) towards substrate-containing model membranes [19,20,21]. This parallel supports the hypothesis that cholesterol may play a functional role in TRPM8 localization and activity, beyond merely serving as a structural component of the membrane.

Several limitations of the study merit discussion, although each is addressed or contextualized. First, TRPM8 is natively a tetrameric ion channel, whereas our purification yields mixtures of predominantly monomeric proteins. While tetramerization may influence membrane interactions in vivo, our experiments demonstrate that even the monomer shows selective association with cholesterol-containing membranes, indicating that the intrinsic protein–lipid recognition is already present at the monomeric level. This is further supported by studies such as Zakharian et al., where purified TRPM8 prepared under similar conditions retains functional activity, suggesting that monomeric TRPM8 can adopt native-like structural features relevant for membrane association.

Second, concerns about protein folding and functional competence are valid. We did not directly assess folding using structural or ligand-binding assays. However, Western blot analysis confirms the presence of TRPM8 epitopes, and the FRET-based interaction with cholesterol-containing membranes demonstrates functional relevance of the purified protein in a defined system. The combination of our data with the literature shows that the preparation preserves key structural and functional properties necessary for biophysical characterization.

Third, fraction 2 from SEC is enriched in monomeric TRPM8 but may contain minor fragments or contaminants. Although Ni-affinity chromatography was initially attempted, yield limitations prevented its use for downstream experiments (Supplementary Material, Figure S2). Nevertheless, native gel electrophoresis analysis confirms that TRPM8 is the major component, and the reproducible preference for cholesterol-containing LUVs indicates that the observed interactions reflect specific protein behavior rather than nonspecific effects. SEC-based molecular weight estimates, while influenced by the detergent micelle, provide consistent relative sizing to identify enriched monomeric fractions, sufficient for the comparative analyses conducted here.

Fourth, the LUV system represents a simplified model of lipid raft-like domains. While vesicles lack additional cellular components such as proteins or glycolipids, the lipid composition used accurately mimics sphingomyelin/cholesterol ratios found in native rafts. Thus, although absolute extrapolation to cellular membranes requires caution, the observed cholesterol-dependent association provides mechanistic insight into the membrane preference of TRPM8.

Finally, the FRET-based measurement reflects an effective membrane-association parameter rather than a precise binding constant. Nonetheless, the method reliably reports enhanced proximity between TRPM8 tryptophans and fluorescent lipids, showing consistent enrichment in cholesterol-containing membranes. This provides robust evidence of selective membrane association, even if direct sterol binding to specific sites is not formally measured.

In conclusion, while each of these limitations could be raised as potential caveats, we have demonstrated either experimentally or through literature support that the purified TRPM8 behaves consistently with native-like membrane affinity. Our study therefore provides novel quantitative insight into TRPM8–membrane interactions, highlighting cholesterol-dependent enrichment and establishing a minimal model system for future investigations into structural and functional determinants of TRPM8 in more complex, tetrameric assemblies.

## 5. Conclusions

In this study, we successfully established a robust experimental framework for the expression, purification, and functional characterization of the human TRPM8 ion channel. By employing a bacterial expression system and optimizing solubilization conditions with DDM detergent, we obtained purified TRPM8 protein suitable for downstream biochemical and biophysical analyses. The use of HPLC-SEC allowed us to isolate distinct protein fractions, among which the monomeric form was identified and further investigated for its membrane-binding properties.

Through FRET-based assays using synthetic lipid vesicles, we demonstrated that TRPM8 exhibits a marked preference for cholesterol-containing membranes. This selective affinity, quantitatively supported by significantly lower L_1/2_ values in the presence of cholesterol, suggests that cholesterol plays a critical role in modulating TRPM8’s membrane association. Our findings provide the first direct in vitro evidence of TRPM8–cholesterol interaction using purified protein and model membranes, reinforcing the hypothesis that cholesterol-rich microdomains, such as lipid rafts, may serve as functional platforms for TRPM8 localization and activity.

Moreover, the observed binding behavior of TRPM8 parallels that of other membrane-associated enzymes, such as lipoxygenase and FAAH, which also display substrate-like affinity for cholesterol-enriched environments. This analogy supports the broader concept that lipid composition, and cholesterol in particular, is not merely a structural component but an active determinant of protein function and spatial organization within the membrane.

Altogether, our work not only advances the biochemical understanding of TRPM8 but also establishes a valuable model system for exploring lipid–protein interactions. These insights may inform future studies aimed at elucidating the structural basis of cholesterol sensitivity in TRPM8 and could contribute to the development of novel therapeutic strategies targeting lipid-dependent modulation of ion channels.

## Figures and Tables

**Table 1:** HPLC-SEC molecular weight determination as a function of peak retention time (R_t_).

Fraction	R_t_ (min)	MW (kDa)
1	15.95	145
2	16.55	109
3	17.42	72
4	18.23	49

**Table 2:** Binding affinity of TRPM8 fractions to synthetic LUVs with and without cholesterol (mean and standard deviation of triplicates).

Fraction	Cholesterol-Containing LUVs [L]_1/2_ (μM) ^A^	LUVs Without Cholesterol [L]_1/2_ (μM) ^A^	*p*-Value
1	22.6 ± 5.2	26.5 ± 5.4	0.419
2	20.6 ± 4.8	62.3 ± 15.1	0.0315 *
3	67.0 ± 15.9	82.7 ± 20.0	0.350

^A^ For the definition of [L]_1/2_, see Equation (1). * *p* < 0.05.

## Data Availability

The raw data supporting the conclusions of this article will be made available by the authors on request.

## Supplementary Materials

The following supporting information can be downloaded at: https://www.mdpi.com/article/10.3390/life16030392/s1, Figure S1, from left to right: native gel electrophoresis silver nitrate staining of native TRPM8; ponceau red staining of the same sample after membrane transfer, molecular weight markers, molecular weight chart and Western blot of the same sample. Proteins (50 μg/well) were electrophoretically separated with 6% polyacrylamide gel electrophoresis (NATIVE PAGE) using Tris-glycine buffer without SDS at a constant voltage of 200 V. Precision Plus Protein™ Kaleidoscope™ (Bio-Rad) were used as protein standards; Figure S2, SDS-PAGE analysis of TRPM8 purification attempts using affinity chromatography Ni-NTA His Bind® Resin (Merck). The column was equilibrated with 6 mL of equilibration buffer (20 mM Tris-HCl, 500 mM NaCl, 5 mM imidazole, 0.5% DDM, pH 7.9). hTRPM8 solubilized with LCB buffer and diluted with three volumes of equilibration buffer was loaded onto the column. After washing the protein was eluted from the column by applying equilibration buffer containing 250 mM imidazole. From left to right: molecular weights chart, molecular weights standards (Precision Plus Protein™ Kaleidoscope™ (Bio-Rad)), eluted protein. Coomassie blue staining.
