# Supplementary material for: HPLC Purification of TRPM8 and Experimental Confirmation of Its Cholesterol Affinity on Synthetic Lipid Raft-like Models
Source: Life (Basel). 2026 Feb 28;16(3):392. doi: 10.3390/life16030392 (PMC13028388; doi:10.3390/life16030392)
Supplement: Supplementary file 1 [file life-16-00392-s001.zip › life-4080982-supplementary.pdf]

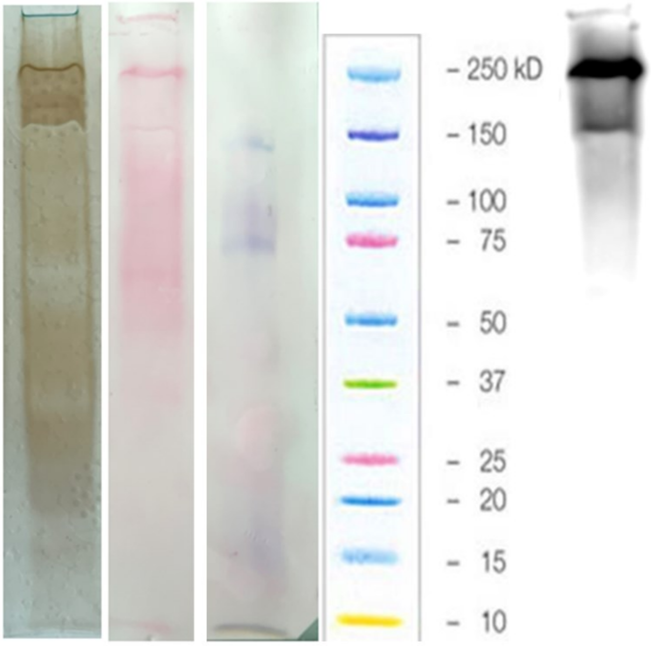

Figure S1. from left to right: native gel electrophoresis silver nitrate staining of native TRPM8; ponceau red staining of the same sample after membrane transfer, molecular weight markers, molecular weight chart and western blot of the same sample. Proteins (50 µg/well) were electrophoretically separated with 6% polyacrylamide gel electrophoresis (NATIVE PAGE) using Tris-glycine buffer without SDS at a constant voltage of 200 V. Precision Plus Protein™ Kaleidoscope™ (Bio-Rad) were used as protein standards.

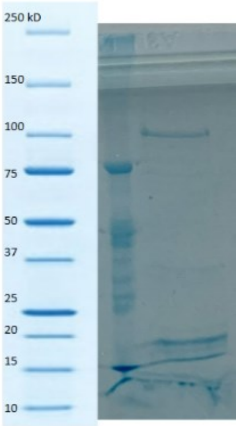

Figure S2. SDS-PAGE analysis of TRPM8 purification attempts using affinity chromatography Ni-NTA His Bind® Resin (Merck). The column was equilibrated with 6 mL of equilibration buffer (20 mM Tris-HCl, 500 mM NaCl, 5 mM imidazole, 0.5% DDM, pH 7.9). hTRPM8 solubilized with LCB buffer and diluted with three volumes of equilibration buffer was loaded onto the column. After washing the protein was eluted from the column by applying equilibration buffer containing 250 mM imidazole. From left to right: molecular weights chart, molecular weights standards (Precision Plus Protein™ Kaleidoscope™ (Bio-Rad)), eluted protein. Coomassie blue staining.
